# Integrating Ultrabright Polymer Dots and Stereo NIR‐II Imager for Assessing Anti‐Angiogenic Drugs in Oral Cancer Model

**DOI:** 10.1111/jcmm.70324

**Published:** 2025-01-05

**Authors:** Bo‐Han Huang, Fang‐Yu Li, Shih‐Po Su, Chiung‐Tong Chen, Kuo‐Wei Chang, Muh‐Hwa Yang, Min‐Chieh Chen, Huihua Kenny Chiang, Yang‐Hsiang Chan, Yi‐Jang Lee

**Affiliations:** ^1^ Department of Biomedical Imaging and Radiological Sciences National Yang Ming Chiao Tung University Taipei Taiwan; ^2^ Department of Biomedical Engineering National Yang Ming Chiao Tung University Taipei Taiwan; ^3^ Institute of Biotechnology and Pharmaceutical Research National Health Research Institutes Zhunan Taiwan; ^4^ Department of Dentistry National Yang Ming Chiao Tung University Taipei Taiwan; ^5^ Institute of Clinical Medicine National Yang Ming Chiao Tung University Taipei Taiwan; ^6^ Cancer and Immunology Research Center National Yang Ming Chiao Tung University Taipei Taiwan; ^7^ Department of Oncology Taipei Veterans General Hospital Taipei Taiwan; ^8^ Department of Applied Chemistry National Yang Ming Chiao Tung University Hsinchu Taiwan; ^9^ Biomedical Engineering Research and Development Center National Yang Ming Chiao Tung University Taipei Taiwan; ^10^ Department of Medicinal and Applied Chemistry Kaohsiung Medical University Kaohsiung Taiwan; ^11^ Center for Emergent Functional Matter Science National Yang Ming Chiao Tung University Hsinchu Taiwan

**Keywords:** 3D NIR‐II fluorescence imaging system, anti‐angiogenic agents, BPR0C261, HIF‐1α, polymer dots, tumour vascularity

## Abstract

The development of efficient platforms for the evaluation of anti‐angiogenic agents is critical in advancing cancer therapeutics. In this study, we exploited an ultrabright semiconducting polymer dots (Pdots) integrating with a three‐dimensional (3D) near‐infrared‐II (NIR‐II) fluorescence imaging system designed to assess the efficacy of potent anti‐angiogenic agents PX‐478 and BPR0C261 in an oral squamous cell carcinoma (OSCC) tumour model, which depends on angiogenesis for dissemination. PX‐478, a hypoxia‐inducible factor‐1α (HIF‐1α) inhibitor, and BPR0C261, a microtubule‐disrupting agent, were administrated into tumour‐bearing mice established using murine MTCQ1 tongue cancer cells through intraperitoneal injection and oral gavage, respectively. Our findings showed that PX‐478 and BPR0C261 significantly inhibited tumour growth and extended the life span of tumour‐bearing mice without decreasing the body weights. The Pdots‐based NIR‐II vascular imaging demonstrated that the tumour vascularity was suppressed by PX‐478 and BPRC0261. Accordingly, the excised tumours treated with anti‐angiogenic agents showed less blood vessels than that treated with vehicles. The expression of endothelial markers CD31 was also found to be reduced in tumours treated with PX‐478 and BPRC0261 using immunohistochemical (IHC) staining and Western blot analysis. Furthermore, PX‐478 could suppress the expression of HIF‐1α and vascular endothelial growth factor‐A (VEGF‐A), but BPRC0261 only suppressed VEGF‐A. Taken together, this innovative 3D NIR‐II imaging system combining the biocompatible Pdots with unique optical specificity enables non‐invasive, real‐time monitoring the efficacy of anti‐angiogenic compounds.

## 1 Introduction

Tumour angiogenesis refers to the formation of new blood vessels that supply nutrients and oxygen to tumours and facilitate their growth and metastasis. The process of tumour angiogenesis involves complex signalling molecules and cellular interactive events [1]. Hypoxia‐inducible factor‐1α (HIF‐1α), a pivotal transcription factor responsible for triggering tumour angiogenesis, is believed to be stabilised in tumours that commonly outgrow their existing blood supply and lead to hypoxia [2, 3]. HIF‐1α mediates the upregulation of several pro‐angiogenic factors, such as vascular endothelial growth factor (VEGF) [4, 5], fibroblast growth factor (FGF) [6], platelet‐derived growth factor (PDGF) [7] and angiopoietins [4, 8]. These factors further activate endothelial cells to proliferate and migrate towards the source of the angiogenic signals. Anti‐angiogenic drugs, such as bevacizumab and sorafenib, have been demonstrated to starve the tumour by inhibiting blood vessel formation via inhibition of VEGF and tyrosine kinase signalling, respectively [9, 10]. Despite targeting angiogenesis is an important strategy for cancer therapy, tumour can develop resistance to anti‐angiogenic therapy through various mechanisms [11]. Hence, development of more anti‐angiogenic compounds remains striving. A promising approach should be critical to evaluate the effects of potent drugs on inhibition of tumour vessels in vivo.

Semiconducting polymer dots (Pdots) can attain distinctive optical properties by molecularly engineering their π‐conjugated chemical structures [12]. Numerous literatures have demonstrated that Pdots are promising candidates for a wide range of biomedical applications due to their exceptional characteristics, including unique high‐fluorescence quantum yield and efficient light‐harvesting properties, outstanding photo/colloidal stability, minimal cytotoxicity and easy surface functionalization [13, 14, 15, 16, 17, 18, 19, 20, 21, 22, 23]. Molecular engineering of Pdots has been reported to emit the second near‐infrared (NIR‐II) fluorescence window (1,000–1,700 nm) via modified donor–acceptor–donor (D‐A‐D)‐based scaffolds with long conjugated backbones that can be utilised for stereo tumour mapping in vivo [24]. Visualisation of Pdots generated NIR‐II fluorescence in living organisms requires a specific three‐dimensional (3D) NIR‐II imaging system that is suitable for the detection of systemic blood vessel with 0.6 and 5 mm‐depth resolution and 0.15 mm spatial resolution after intravenous injection [25]. The compact sizes of Pdots allow them to passively accumulate in tumour lesions through the enhanced permission and retention (EPR) effect [21]. However, imaging of vasculatures encompassing tumours can be achieved at shorter time of administration. Exploitation of Pdots integrated with 3D NIR‐II imaging system is promising for the evaluation of anti‐angiogenic compounds on inhibition of tumour angiogenesis and progression.

As tumour angiogenesis is mainly initiated by HIF‐1α/VEGF signalling, inhibition of HIF‐1α is likely to influence the tumour vasculatures. PX‐478 (*S*‐2‐amino‐3‐[4′‐*N*,*N*,‐bis(chloroethyl)amino]phenyl propionic acid *N*‐oxide dihydrochloride) is a small molecule that can selectively suppress the expression and deubiquitination of HIF‐1α [26]. Several lines of evidence have shown that PX‐478 exhibits anti‐tumour activity in various cancers in vitro and in vivo [26, 27, 28, 29]. PX‐478 is also the first HIF‐1α inhibitor entering Phase I clinical trial [30]. In addition to the HIF‐1α inhibitor, the microtubule‐disrupting agent named BPR0C261 is an analogue of *N*‐heterocyclic indolyl glyoxylamides exhibiting antimitotic, anti‐angiogenic, and radiosensitive capacities [31, 32]. BPR0C261 has been claimed as an oral administrable antitumour compound that possesses a broad spectrum of anticancer activity [33]. Although PX‐478 and BPR0C261 are speculated to inhibit tumour angiogenesis through different pathways, a direct evidence such as vessel imaging remains scarce.

Oral squamous cell carcinoma (OSCC) is the most common type of head and neck squamous cell carcinoma (HNSCC). Enhancement of angiogenesis correlates to the invasion and metastasis of OSCC and accounts for the 5‐year survival rates lower than 50% after conventional diagnosis and therapy [34]. OSCC is located in highly vascularized tissues, but the anti‐angiogenic compounds specifically used for OSCC remain unavailable [35, 36]. Whether inhibition of HIF‐1α and angiogenesis would lead to prominent anti‐tumour effects on OSCC are interesting to be investigated.

In this study, we established an OSCC syngeneic tumour model to evaluate the tumour vascularity before and after the treatments of PX‐478 and BPR0C261. We showed that both compounds raised similar effects on inhibiting the tumour growth. PX‐478 displayed better tumour vascular suppression than BPR0C261 using NIR‐II‐emitted Pdots, and this result was consistent with the responses of VEGF expression in tumours distinctly treated with these compounds. Our data suggest that the semiconducting Pdots combining a 3D NIR‐II fluorescence imaging system would be a convincible approach for the evaluation of potent anti‐angiogenic drugs for cancer therapy.

## 2 Materials and Methods

### 2.1 Preparation of Pdots

Preparation of Pdots involved thoroughly mixing 200 μL of a solution containing the semiconducting polymer (1 mg/mL in tetrahydrofuran (THF)) and 0.04 mg of (1,2‐distearoyl‐sn‐glycero‐3‐phosphoethanolamine with conjugated methoxyl poly(ethylene glycol)) (mPEG‐DSPE‐2000, *M*_w_ = 2,000) in 2.0 mL of THF. The polymer‐containing THF solutions were added to 4 mL of water while subjecting the mixture to intense sonication. The hydrophobic tails of lipid facilitate the encapsulation of hydrophobic polymers, while the hydrophilic heads functionalized with carboxylic acid groups oriented outward into the aqueous environment. Subsequently, the THF solution was swiftly injected into a water solution, followed by ultrasonication and solvent evaporation by applying reduced pressure at room temperature, resulting in the formation of Pdots. The resulting solution of Pdots was then filtered using a 0.2‐μm cellulose acetate syringe filter and became ready for utilisation. This freshly prepared Pdots solution exhibited optical stability for a minimum of two weeks at room temperature when stored in darkness.

### 2.2 In Vivo Near‐Infrared II (NIR‐II) Fluorescence Imaging

Application of Pdots for imaging of tumour vascularity in vivo was referred to a previous report with modification [25]. In brief, 0.25 mg/mL of Indocyanine green (ICG) or 5 mg/mL of Pdots (*E*_m_: 1,000 nm) were intravenously injected into tumour‐bearing mice and then immediately anaesthetised with 2% isoflurane in the custom‐made NIR‐II fluorescence imaging system conducted with different long‐pass (LP) filters under the 793‐nm laser irradiation. The images were acquired using a cooled InGaAs camera (NIRvana 640, Princeton Instruments; 640 × 512 pixels, response 900–1,700 nm) and a 35‐mm C‐mount zoom lens (LM35HC‐SW, Kowa, Tokyo, Japan). This instrument is located and operated in Prof. Huihua Kenny Chiang's lab, Department of Biomedical Engineering, National Yang‐Ming Chiao‐Tung University, Taipei, Taiwan.

### 2.3 Cell Lines and Chemicals

Mouse tongue carcinoma MTCQ1 cells were obtained by 4‐nitroquinoline 1‐oxide treatment and followed by transduction of firefly luciferase (fLuc) reporter gene [37]. Cells were maintained in Dulbecco's modified Eagle medium (GIBCO Invitrogen Inc., Carlsbad, CA, USA), supplemented with 10% fetal bovine serum (HyClone Thermo, Waltham, MA, USA), 50 μg/mL of penicillin/streptomycin (Sigma‐Aldrich Co., St. Louis, MO, USA), 2 mM of l‐glutamine (Sigma‐Aldrich Co., St. Louis, MO, USA), incubated at 37°C in a humidified incubator with 5% CO_2_ and passaged every two days. PX‐478 (TargetMol Inc., Wellesley Hills, MA, USA) was dissolved in 0.9% NaCl. BPR0C261 was synthesised in the Institute of Biotechnology and Pharmaceutical Research, National Health and Research Institute, Zhunan, Taiwan [31]. It was dissolved in DMSO/cremophor EL (polyoxyl 35 castor oil from BASF, Ludwigshafen, Germany)/water (5/20/75%: v/v/v) for gavage.

### 2.4 Animal Model

For xenograft tumour model, the male nude mice (6‐week‐old) were purchased from National Laboratory Animal Center, Taipei, Taiwan. MTCQ‐1 cells (1 × 10^6^) were resuspended in 100 μL of OPTI‐MEM (Sigma‐Aldrich Inc., St., Louis, MO, USA) and then subcutaneously injected into the right hindlimb of nude mice. The tumour‐bearing mice were separated to i.p. injected with PX‐478 (100 mg/kg/days) for five days or orally gavage with BPR0C261 (200 mg/kg/2 days) for three times. The body weights of mice were measured daily. The tumour volume was caliperly measured and calculated using the following formula: volume = (width^2^ × length)/2 daily after tumour growth was palpable. The experiments were terminated when tumour size reached 2,000 mm^3^. The animal study was approved by Institutional Animal Care and Use Committee (IACUC) of National Yang Ming Chiao Tung University (IACUC number:1100509).

### 2.5 In Vivo Optical Imaging

The IVIS Lumina X5 system (Perkin‐Elmer Inc., Billerica, MA, USA) was used to monitor the expression of fLuc reporter gene in tumours formed by MTCQ1 cells. Mice were i.p.‐injected with 150 mg/kg d‐luciferin (Calliper Co., Hopkinton, MA, USA) and then anaesthetised using 2% isoflurane. The mice were then placed in the imaging system with anesthetisation for data acquisition after 15 min. The regions of interest (ROIs) are semi‐quantified as photons/s/cm^2^/sr. Data quantification will be analysed using the bundled Living Imaging Software (Ver. 4.7).

### 2.6 Blood Vessel Analysis

Image processing and vascular density measurements were conducted using a custom‐made computer program developed in MATLAB software (The MathWorks, Natick, MA, USA). The vascular density was computed utilising the proportion method. Initially, a thresholding algorithm was employed to generate a binary image from the NIR‐II fluorescence vasculature image within ROI, denoted by red square. Subsequently, this algorithm was utilised to filter out the background noise and extract the blood vessels within the ROI. Finally, the vascular density was calculated within the ROI using the formula: vascular density = vessel area/total area × 100%.

### 2.7 Haematoxylin and Eosin (H&E) Staining

Tumour sections were prepared for formalin‐fixed paraffin‐embedded tissues and selected according to the morphological catalogue of the diagnosis. The sections were dewaxed in an oven at 60°C, de‐paraffinised by xylene and followed by rehydration in an alcohol solution. The tissue sections were stained with haematoxylin and eosin. The slides were visualised and images were acquired using an optical microscope with a digital camera (Olympus, Center Valley, PA, USA).

### 2.8 Immunohistochemistry (IHC) Staining

Tumours resected from tumour‐bearing mice were rinsed with PBS and fixed in 4% paraformaldehyde with gentle shaking at 4°C overnight. The paraffin‐embedded tissue sections were de‐paraffinised in xylene (Sigma‐Aldrich, St. Louis, MO, USA) for 30 min followed by rehydration in graded ethanol and finally in PBS. The tissue sections were blocked in goat serum containing 5% H_2_O_2_ and incubated with primary antibody for 1.5 h followed by the horseradish phosphatase‐conjugated secondary antibody for another 1 h. The tissue sections were immersed in 3′,3′‐diaminobenzidine (Dako Denmark A/S Produktionsvej 42 DK‐2600 Glostrup Denmark) for development and then counterstained with haematoxylin. The antibodies used for IHC will include antibodies against VEGF‐A (GeneTex Inc. Alton Pkwy Irvine, CA, USA), CD31 and HIF‐1 α antibodies (Abcam, Cambridge, MA, USA). The images were acquired using the optical microscope as mentioned earlier.

### 2.9 Western Blot Analysis

Tumours were collected from the tumour‐bearing mice and lysed in T‐Pro RIPA Lysis Buffer (T‐Pro Biotechnology) containing 1% protease inhibitor cocktail (Sigma‐Aldrich Co., St. Louis, MO, USA). Protein lysates (30 μg) were run on 8%–12% SDS–PAGE, electro‐transferred to nitrocellulose membrane, blocked and incubated with antibody as reported previously [38]. The primary antibodies were anti‐VEGF‐A, anti‐fibroblast growth factor receptor‐1 (FGFR‐1), anti‐CD31, anti‐HIF‐1α, anti‐β‐actin (Abcam, Cambridge, MA, USA) and anti‐glyceraldehyde‐3‐phosphate dehydrogenase (Invitrogen Inc., Carlsbad, CA, USA).

### 2.10 Statistical Analysis

Experimental data were presented as the mean of three independent experiments ± standard deviation. Data were analysed using Student's *t*‐test or one‐way ANOVA (for animal experiments). The software used for statistical analysis was GraphPad Prism V.9.0 (GraphPad Software, Boston, MA, USA). When *p* < 0.05, the results were regarded as statistically significant.

## 3 Results

### 3.1 Preparation and Application of Semiconducting Pdots for Imaging of Tumour Vascularization In Vivo

**FIGURE 1:**
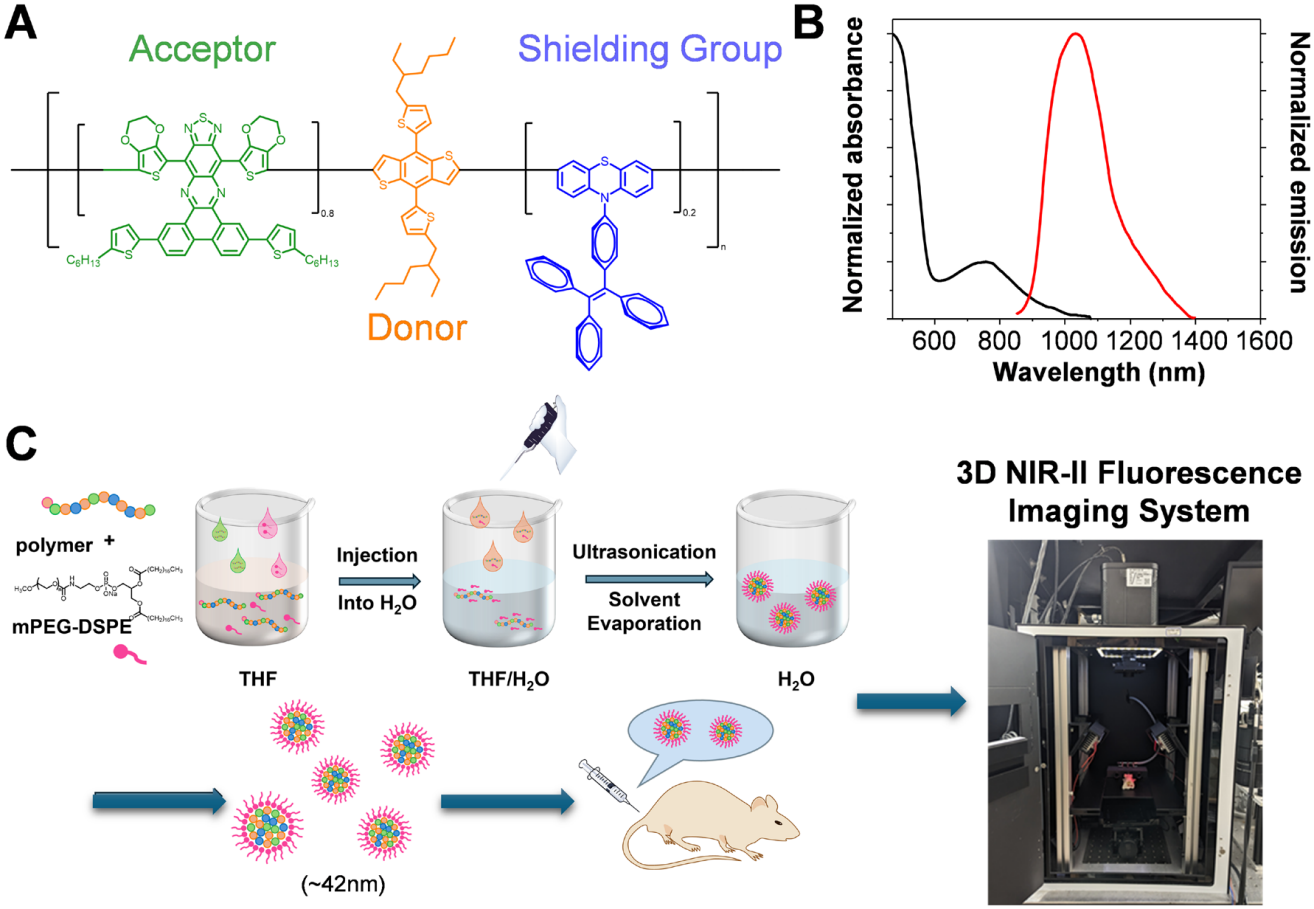
The chemical and physical property of Pdots: (A) The chemical structure of NIR‐II semiconducting polymer dots (Pdots) with a D‐A‐D‐based backbone containing a shielding group; (B) the light spectrum of absorption and emission of Pdots; (C) the procedure of Pdots preparation and administration of mouse. In brief, semiconducting polymer and mPEG‐DSPE lipid were mixed well in THF and then coprecipitated in water under vigorous sonication to form peg‐terminated Pdots with an average hydrodynamic diameter of 42 nm. The Pdots were subsequently injected into the mouse intravenously for NIR‐II fluorescence imaging.

A novel structure of Pdots was composed by usingbis(3,4‐ethylenedioxy thiophene)‐[1,2,5]thiadiazolo[3,4‐g]quinoxaline derivative as the low band‐gap acceptor and an alkylthiothiophene‐substituted benzodithiophene as the donor with tetraphenylethene‐bearing phenothiazine as the bulky spacer (Figure 1A). The absorption and emission maxima of this Pdots nanoparticles locate at around 736 and 1,040 nm, respectively (Figure 1B). The broad emission profile in the NIR‐II region is suitable for deep‐tissue imaging using LP filters, such as 1,100 or 1,200 nm LP filters. The optical properties of Pdots were summarised in Table 1. The scheme of Pdots preparation was illustrated in Figure 1C with mPEG‐DSPE as the capping agent by nanoprecipitation preparation method, resulting in the formation of Pdots nanoparticles in pure aqueous solution. The resulting Pdots were ready for intravenous injection into tumour‐bearing mice followed by image acquisition using a 3D NIR‐II fluorescence imager equipped with a 793‐nm continuous‐wave fibre laser and a cooled InGaAs camera (Figure 1C).

**TABLE 1:** The optical properties of the Pdots in aqueous solution.

Copolymers	λ^abs^ (nm)^a^	λ^em^ (nm)^b^	Mean size (nm)^c^	QY (%)^d^	τ (ns)^e^
Pdots	736	1,040	42	1.7	5.0

^a^ Absorption maximum.

^b^ Fluorescence emission maximum.

^c^ Measured by dynamic light scattering (DLS) analysis.

^d^ Quantum yield of fluorescence (1,040 nm).

^e^ Fluorescence lifetime.

### 3.2 Comparison of PX‐478 and BPR0C261 on Suppression of OSCC in a Syngeneic Tumour Model

**FIGURE 2:**
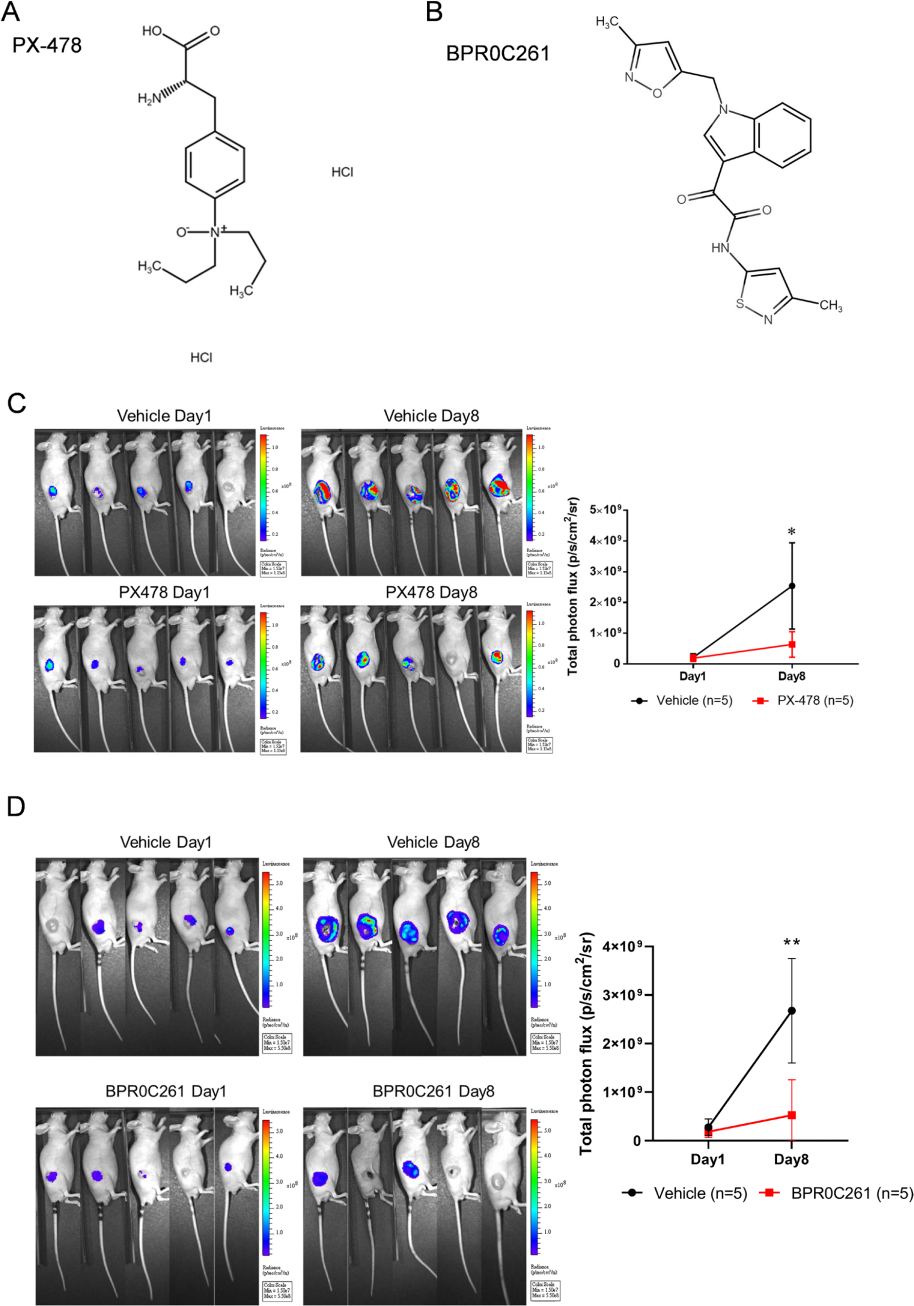
Effects of PX‐478 and BPR0C261 on OSCC syngeneic tumours: (A) and (B) The chemical structures of PX‐478 and BPR0C261, respectively; (C) and (D) detection of tumour activity in tumour‐bearing mice treated with PX‐478 and BPR0C261 using the bioluminescent imaging, respectively. The photon flux at tumour lesions was semi‐quantified and compared between vehicle‐treated and drug‐treated groups at days 1 and 8. *p < 0.05; **p < 0.01.

We first investigated the effects of HIF‐1α inhibitor PX‐478 and microtubule inhibitor BPR0C261 on tumour progression prior to imaging of tumour angiogenesis. The chemical structures of PX‐478 and BPR0C261 were diagrammed in Figure 2A,B, respectively. The tumour‐bearing mice were established using s.c.‐injected murine MTCQ1 tongue squamous cell carcinoma cells. The MTCQ1 cells have been genetically engineered with a luciferase reporter gene (see Materials and Methods section). For PX‐478 treatment, the drug was i.p.‐injected into tumour‐bearing mice with 100 mm^3^ tumours at 100 mg/kg/day for 5 days. The results showed that the bioluminescent signals of PX‐478‐treated tumours were significantly lower than that of vehicle‐treated controls using the optical imaging system after 8 days of treatment (Figure 2C). For BPR0C261, oral gavage of drug at 200 mg/kg/2 days for 3 times was executed, and the similar effect on tumour suppression was also detected (Figure 2D).

### 3.3 Effects of PX‐478 and BPR0C261 on Tumour Growth, Toxicity and Survivals

**FIGURE 3:**
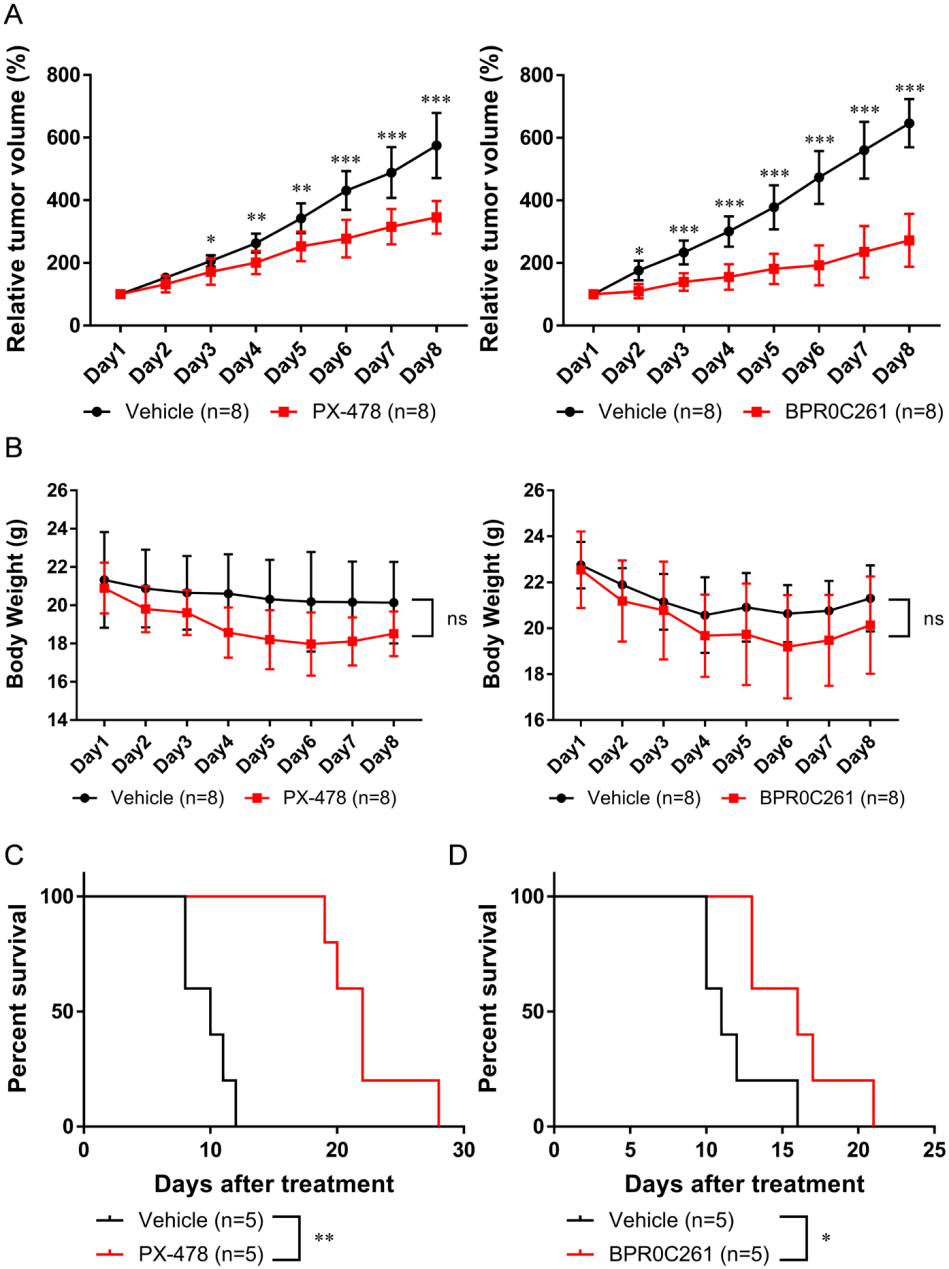
The therapeutic efficacy of PX‐478 and BPR0C261 on OSCC syngeneic tumours: (A) The tumour growth curves of tumour‐bearing mice treated with PX‐478 (left panel) and BPR0C261 (right panel); (B) the body weights of tumour‐bearing mice treated with PX‐478 (left panel) and BPR0C261 (right panel); (C) comparison of the survival rates in tumour‐bearing mice treated with vehicle or PX‐478 and (D) in those treated with vehicle or BPR0C261. *p < 0.05; **p < 0.01; ***p < 0.001.

The tumour growth rates were also tracked and compared between vehicle‐ and drug‐treated groups. Both PX‐478 and BPR0C261 could significantly delay the tumour growth using 2000 mm^3^ as the endpoint of tumour size (Figure 3A). The weights of tumour‐bearing mice were not significantly affected by these compounds under the regimes mentioned earlier (Figure 3B). Moreover, the survival rates of tumour‐bearing mice were increased after they were treated with either PX‐478 or BPR0C261 (Figure 3C). Taken together, the current data demonstrated that the inhibitors of HIF‐1α and microtubule displayed the therapeutic effects on the OSCC tumour model.

### 3.4 Evaluation of PX‐478 and BPR0C261 on Tumour Vascularity by Pdots‐Based NIR‐II Imaging In Vivo

**FIGURE 4:**
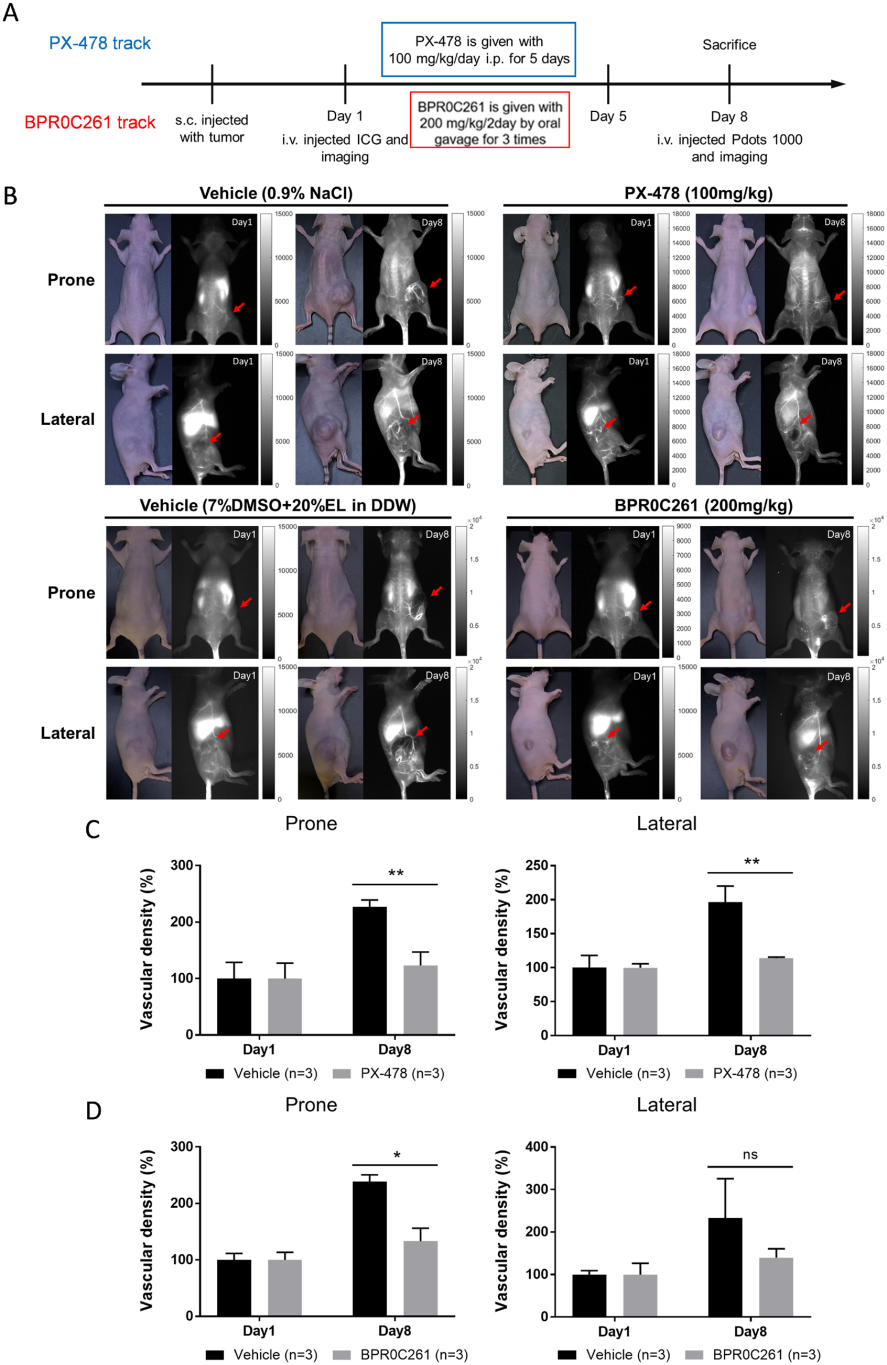
Imaging of tumour vascularity affected by anti‐angiogenic agents using Pdots‐based 3D NIR‐II imaging system: (A) The timeline scheme of MTCQ1 tumour‐bearing mice treated with PX‐478 or BPR0C261 followed by injection of fluorescent agents for vascular imaging; (B) the prone and lateral positions of tumour‐bearing mice imaged under the light field and NIR‐II fluorescence. The tumour lesions were indicated by the red arrows. Images represented the injection of ICG and Pdots at days 1 and 8, respectively; (C) and (D) quantification of vascular density in tumours treated with PX‐478 and BPR0C261 according to the 3D NIR‐II imaging at prone and lateral positions, respectively. *p < 0.05; **p < 0.01; n.s.: Not significant.

We next investigated the effects of PX‐478 and BPR0C261 on the formation of tumour vessels in vivo. The timeline for tumour implantation, drug treatment, and injection of NIR‐II‐emitted agents for vessel imaging was illustrated in Figure 4A. ICG was injected to confirm the initial vascularity in formed tumour at day 1 because ICG is easy to be washed out and appropriate for the followed Pdots treatment at day 8. The results showed that the tumour vessels were conspicuous in vehicle‐treated mouse, but were less detectable in PX‐478 and BPR0C261‐treated mouse using Pdots‐based 3D NIR‐II fluorescence imaging at day 8 (Figure 4B). The vascular density was also quantified and compared in vehicle and drug‐treated groups. For PX‐478, significant reduction of vascular density could be visualised from both prone and lateral imaging position (Figure 3C). For BPR0C261, the similar situation was detected, although the reduced vascular density was not significant at the lateral imaging position (Figure 3D). These results suggest that the effect of potent angiogenic inhibitors on inhibition of tumour vessels can be easily estimated and measured by the Pdots‐based 3D NIR‐II fluorescence imaging system.

### 3.5 Validation of NIR‐II‐Imaged Tumour Vascularity

**FIGURE 5:**
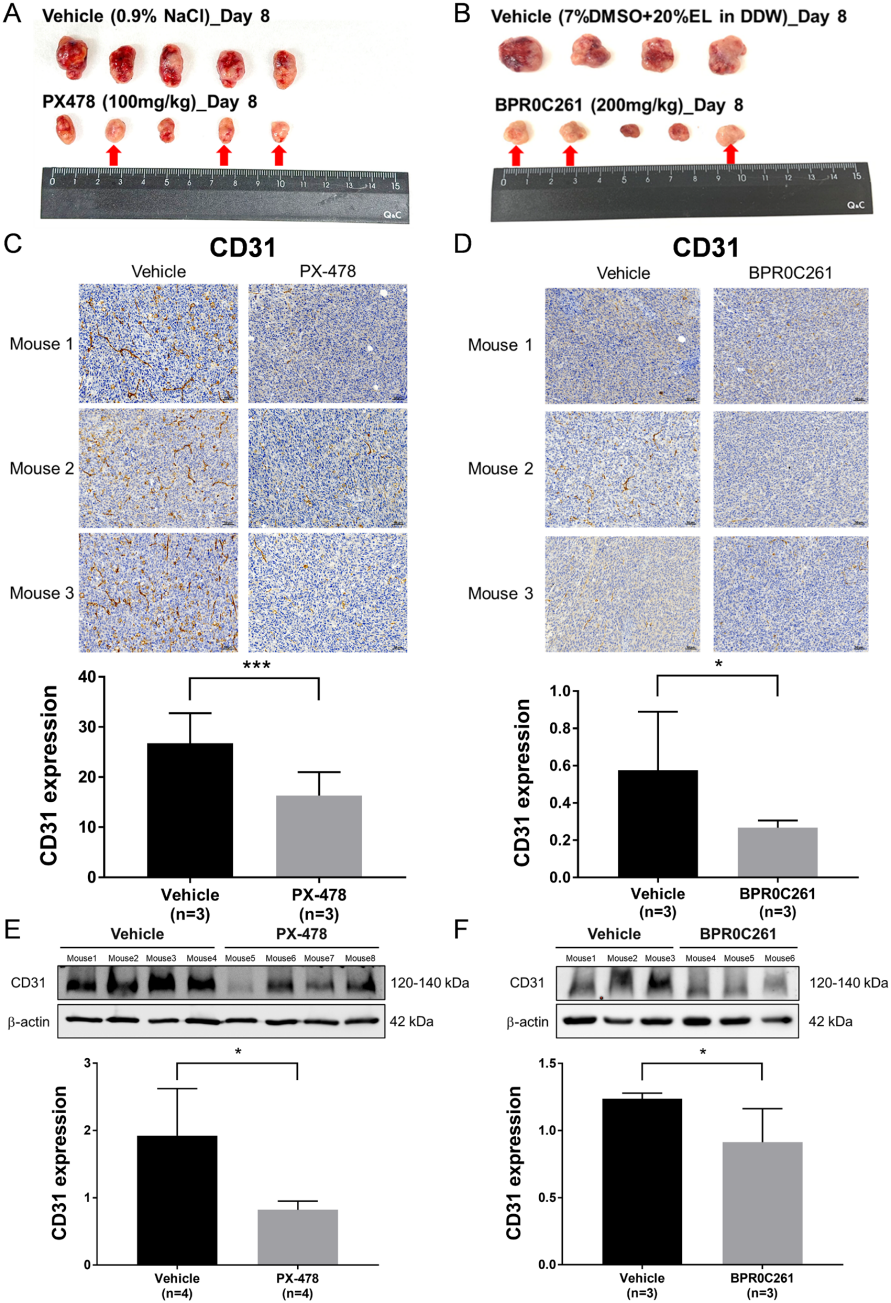
Comparison of PX‐478 and BPR0C261 on tumour vascularity: (A) and (B) The excised MTCQ1 tumours from individual tumour‐bearing mouse treated with or without PX‐478 and BPR0C261, respectively. The red arrows indicate the tumours lacking blood vessels on the surface; (C) and (D) the IHC staining for the expression of CD31 endothelial marker in tumours treated with PX‐478 and BPR0C261, respectively; (E) and (F) Western blot analysis for the expression of CD31 proteins in tumours treated with PX‐478 and BPR0C261, respectively. *p < 0.05; ***p < 0.001.

The implanted MTCQ1 tumours were excised for direct visualisation and IHC staining of CD31 after PX‐478 or BPR0C261 treatment for 8 days. The results showed that untreated tumours were all encompassed by blood vessels, whereas they were barely visible in 3 out of 5 excised tumours treated with drugs (Figure 5A,B). PX‐478 and BPR0C261 also decreased the expression of endothelial cellular marker CD31 in tumour tissues, as demonstrated by IHC staining and quantification (Figure 5C,D). In addition, the Western blot analysis showed that the total CD31 proteins were suppressed in the tumour mass treated with PX‐478 and BPR0C261 (Figure 5E,F).

### 3.6 Effects of PX‐478 and BPR0C261 on the Expression of VEGF‐A in Syngeneic OSCC Tumours

**FIGURE 6:**
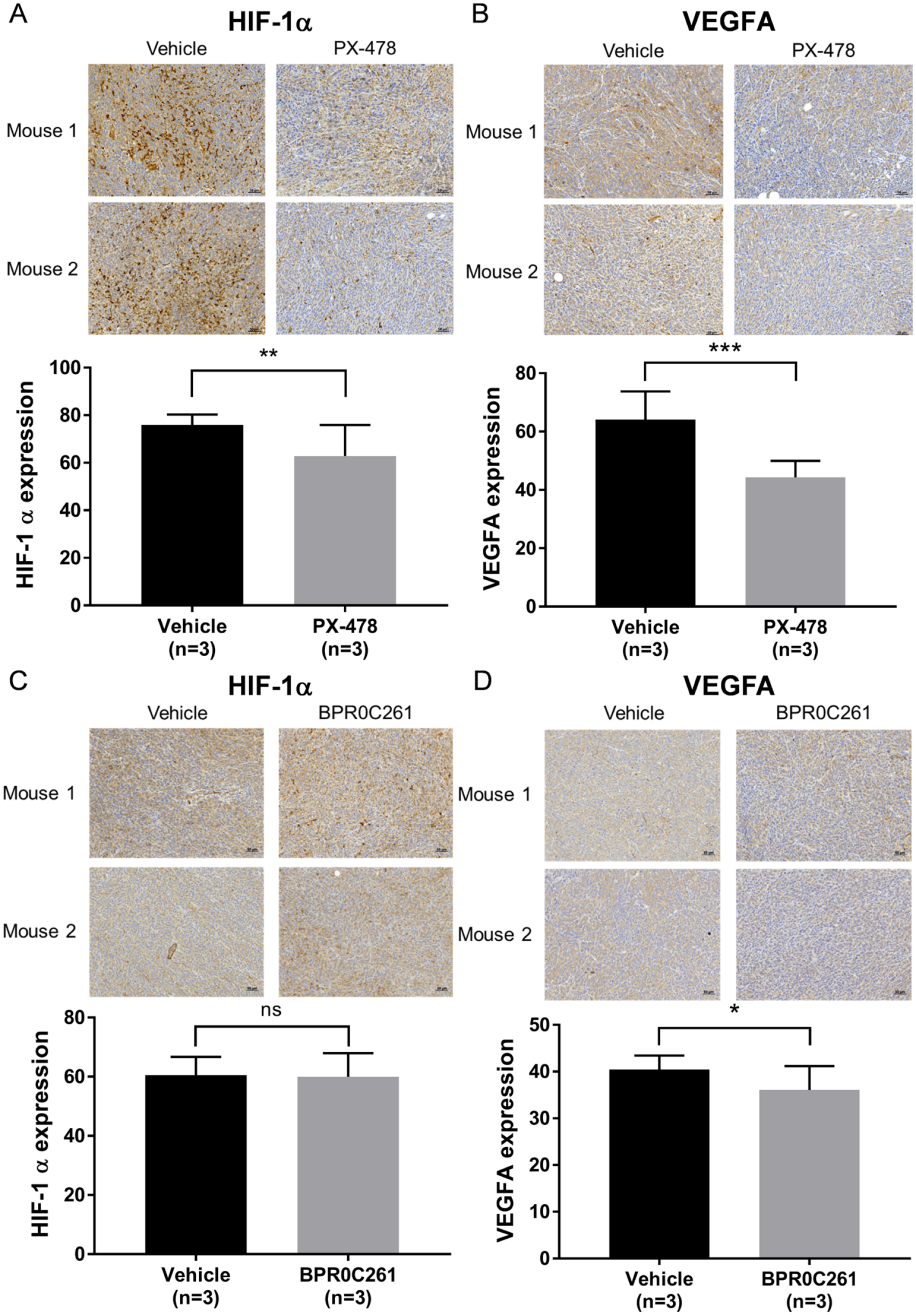
Effects of PX‐478 and BPR0C261 on the expression of HIF‐1α and VEGFA. Comparison of IHC staining for tumours expressed (A) HIF‐1a and (B) VEGFA after the treatment of PX‐478 or left untreated; (C) and (D) the same arrangement was performed for tumours treated with or without BPR0C261. *p < 0.05; **p < 0.01; ***p < 0.001; n.s.: Not significant.

Although PX‐478 and BPR0C261 can suppress the tumour vascularity, it remains unclear if they both suppress HIF‐1α‐mediated angiogenic pathway. We next explored the expression of HIF‐1α and VEGF‐A in MTCQ1 tumours treated with or without drugs. Using IHC staining, the expression of HIF‐1α and VEGF‐A in MTCQ1 tumour sections was significantly suppressed by PX‐478 (Figure 6A,B). On the other hand, HIF‐1α was not affected by BPR0C261, while it inhibited the expression of VEGF‐A in tumour sections (Figure 6C,D). The expression of FGFR‐1 activated by VEGF‐A for angiogenesis [39] was also suppressed by both PX‐478 and BPR0C261 in extracts of MTCQ‐1 tumours (Figure S1). We also utilised CoCl_2_, a hypoxic mimetic compound demonstrated that PX‐478 but not BPR0C261 suppressed the expression of HIF‐1α in cultured MTCQ1 cells, whereas both compounds could inhibit VEGF‐A to different levels (Figure S2). These data suggest that the Pdots‐based 3D NIR‐II‐imaging technique can evaluate the effect of various anti‐angiogenic agents possessing distinct mechanisms to suppress the tumour vascularity.

## 4 Discussion

Imaging tumour angiogenesis is crucial for understanding tumour biology, assessing the effectiveness of anti‐angiogenic therapies, and planning treatment strategies. Molecular imaging of tumour angiogenesis involves using specific imaging techniques to visualise and quantify molecular processes associated with the formation of new blood vessels in tumours. The ultrabright semiconducting Pdots have emerged as a promising tool for NIR‐II because their unique optical and biocompatible properties make them suitable for high‐resolution imaging and precise surgical interventions [40]. There are many synthetic derivatives of NIR‐II‐emitted Pdots [41]. The Pdots used in this study included a shielding group linker phenothiazine with a hydrophobic TPE moiety [24]. The Pdots were also encapsulated with mPEG‐DSPE, the hydrophilic units used for the enhancement of biocompatibility in vivo. Notably, the mean size of Pdots was only 25 nm that is appropriate to imaging small blood vessels surrounding tumours and exhibit the EPR effect that tumours can uptake the nanoparticles [42]. For imaging of tumour vascularity, we did not allow the occurrence of EPR effect by acquiring the data in only 5 min after injection of Pdots. However, our previous study has shown that the EPR effect on tumour lesions could be detected after Pdots were treated for 4 h [43]. Therefore, Pdots have been anticipated as high‐efficiency theragnostic candidates for tumour treatment [44]. Despite current study was designed to use the Pdots‐based NIR‐II imaging as a platform for evaluating the effects of anti‐angiogenic agents, it may consider to conjugate chemotherapeutic agents to Pdots for concomitantly tumour killing in vivo.

Several lines of evidence have shown that Pdots‐based NIR‐II‐imaging approach is ideal for the detection of systemic and tumour blood vessels. Exploitation of this technique for various biomedical purposes is reasonable. For instance, fluorescence image‐guided surgery is a rapid growing discipline in recent year, especially the use of NIR‐II fluorescence for the first‐in‐human liver tumour surgery [45, 46]. A more direct application for Pdots‐based tumour vessel imaging is to evaluate the anti‐angiogenic agents because of non‐invasive, time‐saving and easy‐to‐operative advantages. VEGF and VEGFR are the main targets for design‐related compounds, such as FDA‐approved bevacizumab (Avastin) or sorafenib (Nexavar). Here we selected the HIF‐1α inhibitor PX‐478 because HIF‐1α is a transcription factor of *VEGF* gene. As PX‐478 has never entered the later phase of clinical trial, we are interested in re‐exploring its efficacy on anti‐angiogenesis in vivo. Current data confirmed that PX‐478 showed the inhibitory effect on the growth as well as tumour vascularity in OSCC tumours directly visualised using Pdots‐based NIR‐II vessel imaging. A similar result was also performed in BPR0C261‐treated tumour‐bearing mice. Although BPR0C261 has been reported to exhibit anti‐microtubule and anti‐angiogenesis in various cancer types [31], use of BPR0C261 on OSCC tumour model has not been reported before. These results suggest that the PDots‐based NIR‐II 3D‐imaging system would be an easy and convenient approach for evaluating anti‐angiogenic agents at early stage of development.

Before OSCC tumour model treated with PX‐478 or BPR0C261, the ICG was injected to visualise the original condition of blood vessels encompassing the tumours instead of using Pdots. This is because the washout time of Pdots in tumours was longer than 8 days (data not shown). As mentioned, Pdots could be uptaken by tumours via the EPR effect, so it would interfere the investigation of tumour vessels at the second injection of Pdots. On the contrary, ICG was completely undetectable in vivo at the time of Pdots injection and was suitable for our regime.

In summary, the Pdots‐based 3D NIR‐II fluorescence imaging system is an advanced technique for imaging the blood vascularity in vivo. We have demonstrated that unloaded Pdots can evaluate the effects of anti‐angiogenic agents at a preclinical phase. It is speculated that Pdots would be designed as drug carriers for cancer treatment because of its EPR effect. Notably, utilisation of Pdots for imaging tumour vascularity within minutes makes this technique a quick and reliable platform for selecting anti‐angiogenic compounds for various tumours in vivo.

## Author Contributions

**Bo‐Han Huang:** data curation (equal), methodology (equal). **Fang‐Yu Li:** data curation (equal), methodology (equal). **Shih‐Po Su:** data curation (equal). **Chiung‐Tong Chen:** investigation (equal), resources (equal). **Kuo‐Wei Chang:** resources (equal), supervision (equal). **Muh‐Hwa Yang:** resources (equal), supervision (equal). **Min‐Chieh Chen:** conceptualization (equal), methodology (equal). **Huihua Kenny Chiang:** conceptualization (equal), funding acquisition (equal), writing – review and editing (equal). **Yang‐Hsiang Chan:** conceptualization (equal), funding acquisition (equal), writing – review and editing (equal). **Yi‐Jang Lee:** conceptualization (equal), funding acquisition (equal), writing – original draft (equal).

## Ethics Statement

The animal study was approved by IACUC of National Yang Ming Chiao Tung University (IACUC number: 1100509).

## Consent

The authors have nothing to report.

## Conflicts of Interest

The authors declare no conflicts of interest.

## Supporting information


**Figure S1.** Western blot analysis of VEGF‐A and FGFR‐1 in MTCQ‐1 tumours (tumour‐bearing mice treated with PX‐478 or BPR0C261).

**Figure S2.** Demonstration of PX‐478 on inhibition of the expression of HIF‐1a induced by CoCl_2_: (A) Western blot analysis of HIF‐1a protein expression with the treatment of CoCl_2_ and PX‐478; (B) quantification of the results from Western blot analysis; (C) and (D) the same treatment but use BPR0C261 instead. Each datum was the mean ± SD. *p < 0.05.

## Data Availability

The data that supports the findings of this study are available in the supplementary material of this article.
