# Supplementary figures and images for: Integrating Ultrabright Polymer Dots and Stereo NIR‐II Imager for Assessing Anti‐Angiogenic Drugs in Oral Cancer Model
Source: J Cell Mol Med. 2025 Jan 5;29(1):e70324. doi: 10.1111/jcmm.70324 (PMC11702377; doi:10.1111/jcmm.70324)

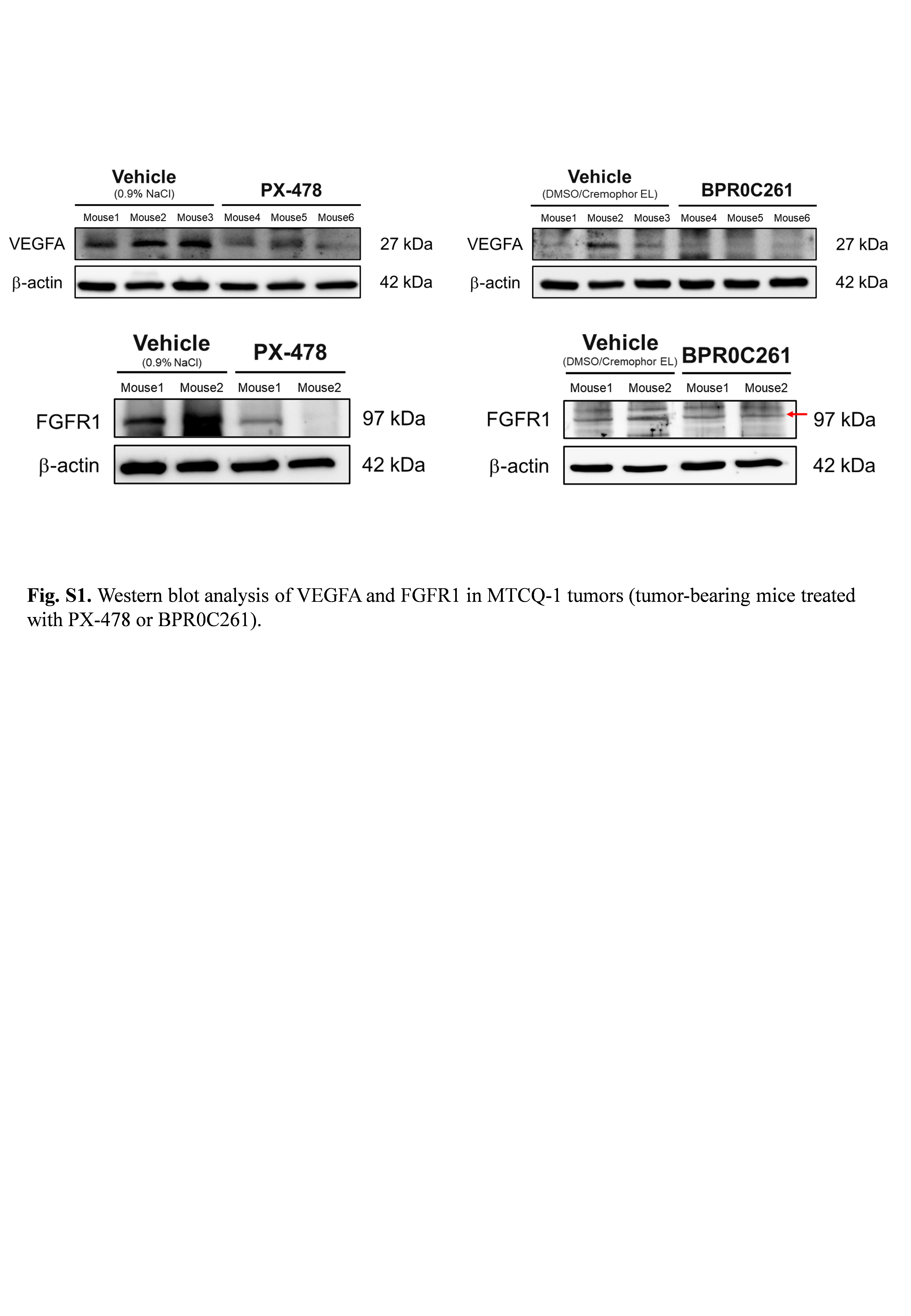

Supplement: Supplementary file 1 — Figure S1. Western blot analysis of VEGF‐A and FGFR‐1 in MTCQ‐1 tumours (tumour‐bearing mice treated with PX‐478 or BPR0C261). [file JCMM-29-e70324-s001.tif]

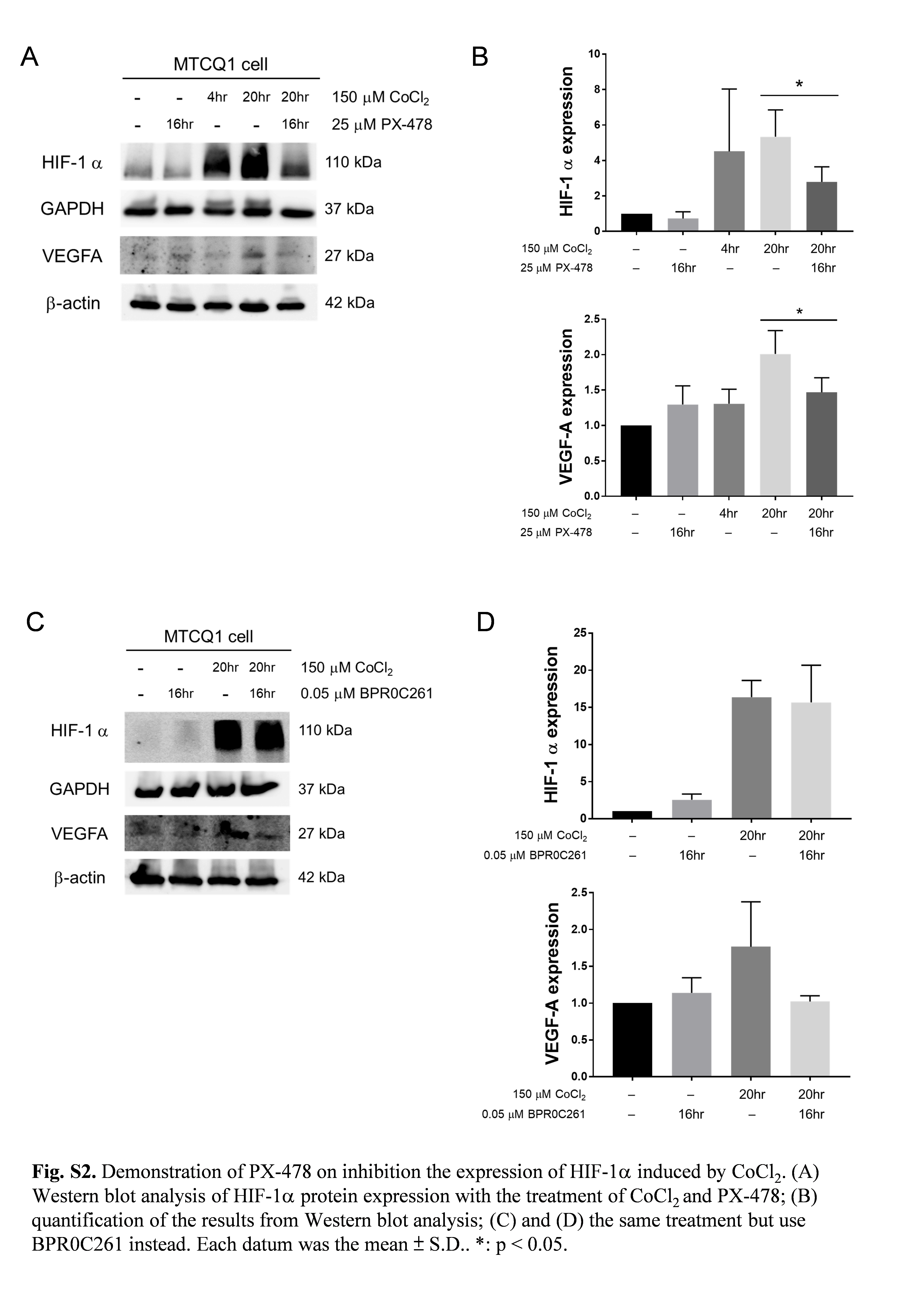

Supplement: Supplementary file 2 — Figure S2. Demonstration of PX‐478 on inhibition of the expression of HIF‐1a induced by CoCl2: (A) Western blot analysis of HIF‐1a protein expression with the treatment of CoCl2 and PX‐478; (B) quantification of the results from Western blot analysis; (C) and (D) the same treatment but use BPR0C261 instead. Each datum was the mean ± SD. *p < 0.05. [file JCMM-29-e70324-s002.tif]
